# Predictive Value of Venous Ductus Doppler in Perinatal Outcomes in Fetal Growth Restriction

**DOI:** 10.7759/cureus.92838

**Published:** 2025-09-21

**Authors:** Branka Cancarevic Djajic, Dragica Draganovic, Tanja Milic-Radic, Suzana Sobot, Bojana Popovic, Jelena Milidragovic, Milan Vjestica, Stefan Bozic, Ljubisa Preradovic

**Affiliations:** 1 Obstetrics and Gynecology, University Clinical Centre of the Republic of Srpska, Banja Luka, BIH; 2 Obstetrics and Gynecology, Faculty of Medicine, University of Banja Luka, Banja Luka, BIH; 3 Anesthesiology and Critical Care, University Clinical Centre of the Republic of Srpska, Banja Luka, BIH; 4 Faculty of Architecture, Civil Engineering and Geodesy, University of Banja Luka, Banja Luka, BIH

**Keywords:** acidosis, apgar score, doppler ultrasound, ductus venosus, fetal growth restriction, perinatal outcome

## Abstract

Introduction

Fetal growth restriction (FGR) is associated with perinatal morbidity, acidosis, and adverse neurological outcomes. While arterial Doppler of the umbilical artery (UA) and middle cerebral artery informs surveillance, ductus venosus (DV) Doppler may better reflect central hemodynamics and impending cardiac decompensation. This study evaluated the predictive value and clinical significance of DV Doppler velocimetry for acid-base status, early postnatal Apgar score (AS), neonatal morbidity, and neurological outcomes in infancy.

Methods

In a retrospective, single-center cohort, pregnancies with and without FGR underwent UA and DV Doppler assessment. DV waveforms were classified as normal or pathological based on the presence of an absent or a reversed A-wave. Neonatal outcomes included umbilical artery blood pH, AS at standard early postnatal intervals, composite neonatal morbidity, and neurological status during the early neonatal period and later infancy. Associations were examined using Fisher’s exact tests, and diagnostic performance was summarized with sensitivity, specificity, positive predictive value, and negative predictive value.

Results

A pathological DV was recorded in 15.4% of fetuses with FGR. In this subgroup, acidosis (pH <7.20) occurred in 100% of cases and was significantly less frequent among fetuses with normal DV findings (p <0.001). At one minute, a low AS (0-3) was present in 75% of newborns with pathological DV, and at five minutes in 25%; all newborns with pathological DV had an AS of ≤7 at both time points, whereas an AS of ≥7 predominated in the normal DV group (p <0.01). Neonatal morbidity was present in 100% of newborns with pathological DV compared with 29.5% in the normal DV group (p <0.001). During the first month, all newborns with pathological DV demonstrated neurological injury on early assessment.

Conclusion

DV Doppler adds clinically actionable information to arterial Doppler in FGR by identifying venous decompensation at a stage when intervention may alter outcome. Incorporating DV evaluation into routine surveillance can trigger timely delivery when venous compromise is present and support expectant management when venous flow remains normal, helping clinicians balance the risks of prematurity against progressive hypoxic-ischemic injury and may inform perinatal care pathways.

## Introduction

Fetal growth restriction (FGR) presents a major challenge in perinatal medicine due to its strong association with adverse outcomes, including perinatal mortality, acidosis, and long-term neurological complications. While Doppler assessment of arterial parameters, such as the resistance index in the umbilical artery (UA) and the middle cerebral artery, is commonly used to monitor FGR, Doppler evaluation of the ductus venosus (DV) provides additional insightS into fetal central hemodynamics and enables early detection of cardiac decompensation, fetal hypoxemia, hypoxia, and metabolic acidosis, which includes neonatal morbidity and mortality as a consequence [1,2].

A pathological flow pattern in the DV, particularly a reversed A-wave, is considered a reliable indicator of terminal fetal decompensation and a predictor of poor perinatal outcomes [3,4]. Because venous flow reflects right heart function, changes in the fetal venous system indicate failure of compensatory mechanisms and the onset of cerebral and myocardial hypoxia -- events that are subsequently mirrored in cardiotocographic (CTG) abnormalities.

Accordingly, the detection of pathological Doppler findings in the arterial system necessitates parallel evaluation of venous Doppler flows [1,2,5]. A reduction in right ventricular contractility typically results in altered DV flow, which often precedes CTG abnormalities stemming from right heart dysfunction and myocardial hypoxia [1].

Changes in arterial flow that suggest blood flow centralization represent a compensatory response to reduced oxygenation and allow for the temporary preservation of oxygen delivery to vital organs [2,6-8]. When these compensatory mechanisms fail, right heart insufficiency develops, followed by CTG abnormalities and hypoxic-ischemic injury. Therefore, the presence of abnormal venous Doppler findings in conjunction with arterial changes signals the need for urgent delivery, as cardiac failure and irreversible central nervous system damage may rapidly ensue [9,10]. The objective of this study was to evaluate the predictive value and clinical significance of DV Doppler velocimetry in pregnancies complicated by FGR, with particular reference to fetal acidosis, low Apgar scores at one and five minutes, neonatal morbidity, and both early (within the first month) and late (within the first six months) neurological outcomes.

## Materials and methods

This retrospective study included 104 pregnant women who were hospitalized at the Department of Perinatology, Clinic for Gynecology and Obstetrics, University Clinical Center, Banja Luka. Participants were evenly divided into two groups: Group A (n = 52), the study group, and Group B (n = 52), the control group. The institutional review board ethics committee approved the study design (approval no. 01-19-277-2/25).

Group A included singleton pregnancies with reliably determined gestational age and a sonographic diagnosis of FGR. Additional inclusion criteria were the absence of congenital malformations or chromosomal abnormalities, completion of a final Doppler examination on the day of delivery, and availability of a known pregnancy outcome. Group B consisted of singleton pregnancies with reliably determined gestational age, no sonographic evidence of FGR, no congenital malformations, and known pregnancy outcomes, including documented neurological assessments at one and six months of age of the neonate. All participants in Group B were at a gestational age of 27 weeks or greater.

All patients underwent Doppler velocimetry of the UA to detect abnormal flow patterns, including absent end-diastolic flow and reversed end-diastolic flow. Doppler assessments of the DV were also performed. These measurements were conducted by one gynecologist-obstetrician. The DV was visualized using color Doppler imaging to identify a cross-shaped vascular structure formed by the abdominal aorta, the inferior vena cava, and the DV. The ultrasound transducer was positioned in a transverse plane at the level of the fetal stomach, allowing identification of the DV, which is located anterior to the aorta and to the right of the stomach’s acoustic shadow. The DV exhibits a characteristic “sparkling” appearance on color Doppler. DV flow was considered pathological if the A-wave was absent or reversed, corresponding to absent or reversed blood flow during atrial systole.

The neonatal condition was evaluated immediately after birth using UA blood pH and Apgar scores at one and five minutes. Prior to the newborn’s first breath, a segment of the umbilical cord measuring 20 to 25 cm was double-clamped, and blood was collected from the UA into heparinized tubes (Type D 551/12.5/200; Radiometer, Copenhagen). The acid-base status of the neonate was analyzed using an ABL2 blood gas analyzer (Radiometer). Umbilical artery pH values were classified into three categories: normal (pH ≥7.25), preacidosis (pH 7.20-7.24), and acidosis (pH <7.20). Apgar scores at one minute were classified as follows: 8 to 10 indicated good vitality, 4 to 7 indicated mild depression or asphyxia, 1 to 3 indicated severe depression or asphyxia, and a score of 0 indicated stillbirth. In this study, there were no stillbirths and no early neonatal deaths.

Neonatal outcomes, including neurological complications during the first six months of life, were monitored and recorded. These outcomes included both early- and late-onset neurological disorders. These included sonographic monitoring of neonatal central nervous system (CNS), presence of hypoxic-ischemic encephalopathy (HIE), periventricular hemorrhage (PVH), periventricular echogenicity (PVE), intracranial hemorrhage (ICH), intraventricular hemorrhage (IVH), and convulsions.

Doppler findings of the DV were compared between the two groups with respect to perinatal outcomes. Based on these results, we determined the clinical significance and predictive value of DV Doppler velocimetry for adverse perinatal events.

Data were statistically analyzed using appropriate non-parametric tests and are presented in tabular, numerical, and percentage formats. Associations between categorical variables were evaluated using Fisher’s exact test, which provided the p-values reported in the results. Because all DV Doppler results in the control group were normal, statistical analysis for that group was not performed. For both groups, the following diagnostic parameters were calculated: sensitivity, specificity, negative predictive value, positive predictive value, and overall reliability.

## Results

Ductus venosus Doppler and umbilical artery blood pH

In the FGR group, DV Doppler findings were normal in most cases, accounting for 84.6% (44 of 52) cases. Within this subgroup, 18.2% (8 of 44) of neonates were acidemic at birth, 13.6% (6 of 44) exhibited preacidosis, and 68.2% (30 of 44) had normal UA blood pH. A pathological DV Doppler was identified in 15.4% (8 of 52), and all neonates with pathological DV Doppler findings (100%) presented with acidosis at birth. Analysis of the type of acidosis showed that three neonates (18.7%) with respiratory acidosis had normal prenatal DV Doppler findings; one newborn (6.2%) with mixed acidosis had a prenatally identified pathological DV Doppler; and among the 12 neonates with metabolic acidosis, seven (58.3%) had a pathological DV, while five (41.6%) had a normal DV Doppler. When the categories of normal and preacidosis pH were combined, Fisher’s exact test demonstrated a highly statistically significant association between DV Doppler status and UA blood pH in the FGR cohort (p <0.001). In the control group, DV Doppler was universally normal, and acidemia was observed in 17.3% of neonates. These findings are summarized in Table 1.

**Table 1:** Ductus venosus Doppler and umbilical artery blood pH in the FGR group and the control group ^a^p-value calculated using Fisher's exact test (FGR group only). DV, ductus venosus; FGR, fetal growth restriction; UA, umbilical artery; NA, not applicable.

Group	DV	UA blood pH			Total, n (%)	p-value^a^
		Normal, n (%)	Preacidosis, n (%)	Acidosis, n (%)		
FGR (n = 52)	Normal	30 (68.2)	6 (13.6)	8 (18.2)	44 (84.6)	<0.001
	Pathological	0 (0.0)	0 (0.0)	8 (100.0)	8 (15.4)	<0.001
Control (n = 52)	Normal	38 (73.1)	5 (9.6)	9 (17.3)	52 (100.0)	NA
Total		68	11	25	104 (100.0)	NA

Apgar scores

At one minute, 61.4% of neonates with normal DV Doppler findings in the FGR group had a normal Apgar score (8-10), while 6.8% had a score between 0 and 3. Among neonates with pathological DV Doppler findings, none had a normal Apgar score; 25% had a score between 4 and 7, and 75% had a score between 0 and 3. After grouping Apgar scores of 0-3 and 4-7, Fisher’s exact test revealed a highly statistically significant association (p = 0.001) between DV Doppler status and the one-minute Apgar score in the FGR cohort. In the control group, most neonates had normal one-minute Apgar scores, although 19.2% scored 4-7 and 1.9% scored 0-3. One-minute Apgar data for both groups are given in Table 2.

**Table 2:** Ductus venosus Doppler and Apgar score at one minute in the FGR group and the control group ^a^p-value calculated using Fisher's exact test (FGR group only). DV, ductus venosus; FGR, fetal growth restriction; NA, not applicable.

Group	DV	Apgar score at one minute			Total, n (%)	p-value^a^
		8–10, n (%)	4–7, n (%)	0–3, n (%)		
FGR (n = 52)	Normal	27 (61.4)	14 (31.8)	3 (6.8)	44 (84.6)	0.001
	Pathological	0 (0.0)	2 (25.0)	6 (75.0)	8 (15.4)	0.001
Control (n = 52)	Normal	41 (78.8)	10 (19.2)	1 (1.9)	52 (100.0)	NA
Total		68	26	10	104 (100.0)	NA

At five minutes, 86.4% of neonates with normal DV flows in the FGR group had a normal Apgar score, whereas 75% of those with pathological DV flows had a score of 4-7. When scores of 0-3 and 4-7 were combined, Fisher’s exact test again showed a highly statistically significant association (p <0.001) between DV Doppler status and the five-minute Apgar score in the FGR cohort. In the control group, 13.5% had a five-minute Apgar score of 4-7, while the remainder had scores indicating good vitality. Five-minute Apgar results for both groups are shown in Table 3.

**Table 3:** Ductus venosus Doppler and Apgar score at five minutes in the FGR group ^a^p-value calculated using Fisher's exact test. DV, ductus venosus; FGR, fetal growth restriction.

DV	Apgar score at five minutes			Total, n (%)	p-value^a^
	8–10, n (%)	4–7, n (%)	0–3, n (%)		
Normal	38 (86.4)	5 (11.4)	1 (2.3)	44 (84.6)	<0.001
Pathological	0 (0.0)	6 (75.0)	2 (25.0)	8 (15.4)	<0.001
Total	38 (73.1)	11 (21.2)	3 (5.8)	52 (100.0)	<0.001

Neonatal outcome (first month)

Among FGR neonates with normal DV Doppler findings, 70.5% experienced a normal neonatal outcome, while 29.5% developed neonatal morbidity. In the control group, 75.0% had a normal outcome and 25.0% experienced morbidity. Respiratory distress syndrome (RDS) was the most frequent morbidity in the control group, affecting 13.4% (7 of 52) of neonates. Fisher’s exact test confirmed a statistically significant association between DV Doppler status and neonatal morbidity within the FGR cohort (p = 0.006). These data are presented in Table 4.

**Table 4:** Ductus venosus Doppler and neonatal outcome in the first month of life in the FGR group and the control group ^a^p-value calculated using Fisher's exact test (FGR group only). DV, ductus venosus; FGR, fetal growth restriction; NA, not applicable.

Group	DV	Neonatal outcome		Total, n (%)	p-value^a^
		Healthy, n (%)	Not healthy, n (%)		
FGR (n = 52)	Normal	31 (70.5)	13 (29.5)	44 (84.6)	0.006
	Pathological	0 (0.0)	8 (100.0)	8 (15.4)	0.006
Control (n = 52)	Normal	39 (75.0)	13 (25.0)	52 (100.0)	NA
Total		70	34	104 (100.0)	NA

Neurological status during the first month

Neurosonography demonstrated a normal neurological status in 70.5% (31 of 44) of FGR neonates with normal DV Doppler findings, whereas 29.5% (13 of 44) had pathological findings, including intracranial hemorrhage, hypoxic-ischemic encephalopathy, and intraventricular hemorrhage. All neonates with pathological DV Doppler findings (100%; eight of eight) had abnormal neurosonographic outcomes. In the control group, 75.0% had a normal neurological status, and 25.0% had pathological findings. Within the FGR cohort, DV Doppler status was highly associated with early-onset neurological disease (p <0.001). Detailed distributions for both groups are shown in Table 5.

**Table 5:** Ductus venosus Doppler and neurological status during the first month of life in the FGR group and the control group ^a^p-value calculated using Fisher's exact test (FGR group only). DV, ductus venosus; FGR, fetal growth restriction; HIE, hypoxic–ischemic encephalopathy; ICH, intracranial hemorrhage; IVH, intraventricular hemorrhage; PVE, periventricular echogenicity; PVH, periventricular hemorrhage; NA, not applicable.

Neurological status	FGR DV normal	FGR DV pathological	FGR total, n (%)	Control DV normal (n = 52)	p-value^a^
Normal	31	0	31 (59.6)	39 (75.0)	<0.001
ICH grade I	0	2	2 (3.8)	1 (1.9)	<0.001
ICH grade I + HIE I	1	0	1 (1.9)	NA	<0.001
ICH grade II	1	0	1 (1.9)	1 (1.9)	<0.001
ICH II–III	0	1	1 (1.9)	NA	<0.001
HIE stage I	2	1	3 (5.7)	NA	<0.001
PVE	1	1	2 (3.8)	NA	<0.001
IVH grade I	3	1	4 (7.7)	3 (5.8)	<0.001
IVH grade I + ICH I	2	0	2 (3.8)	NA	<0.001
IVH I + ICH II–III	1	0	1 (1.9)	NA	<0.001
IVH I + HIE I	0	1	1 (1.9)	NA	<0.001
IVH grade II	1	1	2 (3.8)	2 (3.8)	<0.001
IVH II–III + HIE II	1	0	1 (1.9)	NA	<0.001
Other (PVH, PVH + HIE)	NA	NA	NA	6 (11.5)	NA
Total	44	8	52 (100.0)	52 (100.0)	NA

Late-onset neurological outcomes

During the first six months of life, late-onset neurological sequelae were identified in 62.5% (five of eight) of FGR neonates with abnormal DV Doppler findings and in 18.2% (8 of 44) of those with normal DV findings. In the control group, late-onset neurological conditions were observed in 13.5% (7 of 52). DV Doppler status was significantly associated with late-onset neurological outcomes in the FGR cohort (p = 0.017). Results for both groups are presented in Table 6.

**Table 6:** Late-onset neurological diseases according to ductus venosus Doppler in the FGR group and the control group ^a^p-value calculated using Fisher's exact test (FGR group only). DV, ductus venosus; FGR, fetal growth restriction.

Group	DV	Late-onset neurological diseases		Total, n (%)	p-value^a^
		Yes, n (%)	No, n (%)		
FGR (n = 52)	Normal	8 (18.2)	36 (81.8)	44 (84.6)	0.017
	Pathological	5 (62.5)	3 (37.5)	8 (15.4)	0.017
Control (n = 52)	NA	7 (13.5)	45 (86.5)	52 (100.0)	NA
Total		86	18	104 (100.0)	NA

Specific neonatal morbidities

Analysis of morbidity distribution in the FGR group indicated that among neonates with normal DV Doppler findings, necrotizing enterocolitis (NEC) occurred in two, respiratory distress syndrome in six, hyperbilirubinemia in nine, hyperinsulinemia in two, and perinatal infection in four. In the pathological DV group, five neonates were diagnosed with RDS and one with a perinatal infection. These data are shown in Table 7. Neurological conditions were analyzed separately and are not included in the morbidity table.

**Table 7:** Neonatal morbidity by ductus venosus Doppler status in the FGR group DV, ductus venosus; FGR, fetal growth restriction; NEC, necrotizing enterocolitis; RDS, respiratory distress syndrome.

Morbidity	DV normal, n	DV pathological, n	Total, n
NEC	2	0	2
RDS	6	5	11
Hyperbilirubinemia	9	0	9
Hyperinsulinemia	2	0	2
Perinatal infection	4	1	5

Diagnostic performance of DV Doppler

In the FGR group, DV Doppler demonstrated high specificity (100%) and high positive predictive value for fetal acidosis, low Apgar scores, neonatal morbidity, and early-onset neurological disease; for late-onset neurological outcomes, DV Doppler showed high sensitivity and low specificity. In the control group, the absence of pathological DV Doppler findings yielded very high specificity with zero sensitivity, and the positive predictive value could not be calculated; for late-onset neurological outcomes, DV Doppler showed high sensitivity and zero specificity, and the negative predictive value could not be calculated. The diagnostic performance of DV Doppler velocimetry is summarized in Table 8.

**Table 8:** Predictive value of ductus venosus Doppler for adverse perinatal outcomes in the FGR group and the control group DV, ductus venosus; FGR, fetal growth restriction; NPV, negative predictive value; PPV, positive predictive value; Se, sensitivity; Sp, specificity; P, reliability; NA, not applicable.

Group	Outcome	Sp	Se	NPV	PPV	P
FGR	Acidosis	1.0000	0.5000	0.8182	1.0000	0.8462
	Apgar at 1 min	1.0000	0.3200	0.6136	1.0000	0.6731
	Apgar at 5 min	1.0000	0.5714	0.8636	1.0000	0.8846
	Neonatal outcome	1.0000	0.2759	0.5227	1.0000	0.5962
	Early neurological disease	1.0000	0.3809	0.7046	1.0000	0.7500
	Late neurological disease	0.0769	0.6154	0.3750	0.1818	0.2115
Control	Acidosis	1.0000	<0.0010	0.8269	NA	0.8269
	Apgar at 1 min	1.0000	<0.0010	0.7885	NA	0.7885
	Apgar at 5 min	1.0000	<0.0010	0.9184	NA	0.9184
	Neonatal outcome	1.0000	<0.0010	0.8077	NA	0.8077
	Early neurological disease	1.0000	<0.0010	0.7500	NA	0.7500
	Late neurological disease	<0.0010	1.0000	NA	0.1346	0.1346

## Discussion

The results of this study demonstrate the high predictive value of DV Doppler velocimetry for assessing perinatal outcomes in pregnancies complicated by FGR. Within the first month of life, all newborns with a prenatally detected pathological DV Doppler exhibited neurological abnormalities. In the control group, although no pathological DV Doppler findings were recorded, adverse outcomes were still noted.

These findings are supported by previous research. One study evaluating the diagnostic efficacy of DV Doppler and computerized cardiotocography in early-onset FGR found that their combined use predicted normal neurodevelopmental outcomes at two years of age in 95% of cases [11]. DV Doppler is a sensitive marker of fetal central hemodynamics and offers early detection of cardiovascular compromise. This role is emphasized by several studies indicating that pathological changes in DV flow, especially a reversed A-wave, occur in the terminal phase of fetal adaptation to chronic hypoxia [3,4,12]. These changes typically precede alterations in CTG and the biophysical profile [12].

The high rate of early neurological disorders in the control group (25%) may be attributed to postnatal complications. Respiratory distress syndrome was the most common morbidity, with a prevalence of 13.4%, and may have contributed to postnatal hypoxia and cerebral hypoperfusion. Although occurring after birth, these complications could have resulted in intraventricular hemorrhage and hypoxic-ischemic lesions detected within the first days of life. Infections and hyperbilirubinemia, although classified as mild, may cause sequelae if not optimally treated. Early prematurity or complicated delivery were not fully analyzed in this study, but may represent additional risk factors.

Baschat et al. previously described the emergence of pathological DV findings as a late but critical event in the hypoxic-ischemic cascade, one that signifies impending myocardial failure and a heightened risk of intrauterine demise [10]. Furthermore, DV Doppler has been explored as a potential predictor of necrotizing enterocolitis. In this study, NEC was diagnosed in cases with abnormal DV flow, which aligns with the literature showing that abnormal venous Doppler is associated with an increased risk of NEC in neonates with FGR [13].

Additional studies support the clinical value of DV Doppler. Govender et al. [14] reported that abnormal DV Doppler in pregnancies affected by both FGR and preeclampsia was associated with a six-fold increase in neonatal morbidity. This cohort also exhibited greater perinatal mortality and a higher risk of placental abruption. In the FGR group of the present study, there were eight fetuses (18.2%) with a normal DV Doppler and a UA pH below 7.2, while in the control group, there were nine (17.3%), which implies a notable false negative value (15.38% for the FGR group, and 17.3% for the control group). Conversely, fetuses with abnormal DV findings were significantly more likely to experience low Apgar scores and early neurological injury.

These findings reinforce the importance of incorporating DV Doppler analysis into fetal surveillance protocols. Although arterial Doppler evaluation, such as of the umbilical and middle cerebral arteries, remains essential, the addition of DV assessment provides a critical insight into fetal compensation and cardiovascular stability. This is particularly valuable in determining the timing of delivery in cases of severe or early-onset FGR [15,16]. Studies have consistently shown that abnormal DV Doppler findings are associated with poor perinatal outcomes in early FGR and that absent or reversed A-wave signals represent a decompensated state of fetal distress [17,18].

Limitations

This study has several important limitations. The sample size, particularly the number of fetuses with pathological DV Doppler findings, was relatively small, which may affect generalizability. The study was conducted at a single tertiary center among hospitalized patients, introducing potential referral and selection biases that may limit applicability to broader obstetric populations. Sonographers and clinicians were not blinded to DV Doppler results, and DV status may have influenced clinical management, including the timing of delivery, creating possible indication or incorporation bias. DV assessment was performed at a single time point on the day of delivery rather than longitudinally, so the trajectory of venous changes could not be evaluated. Image acquisition and interpretation were not formally standardized across operators, and interobserver variability was not measured, which may affect reproducibility.

Potential confounders were not fully controlled. The analysis did not adjust for gestational age at delivery, early- versus late-onset FGR, maternal comorbidities such as preeclampsia or diabetes, antenatal corticosteroid exposure, labor management, or mode of delivery, all of which could influence acid-base status, Apgar scores, and neurological outcomes. The control group contained no pathological DV results, which limits estimation of test characteristics in that cohort and may inflate specificity while precluding stable calculation of some predictive values. Statistical inference relied on non-parametric tests without multivariable modeling, so independent associations between DV status and outcomes cannot be established, and causality cannot be inferred.

Outcome ascertainment also has constraints. UA sampling reflects a single post-delivery measurement and may not capture dynamic acid-base changes. Apgar scoring and neurosonographic interpretations are subject to inter-rater variability, and neurological follow-up was limited to six months without standardized neurodevelopmental testing. In addition, neurological diagnoses were analyzed separately from other neonatal morbidities, which may complicate comparisons across outcome categories and underestimate composite morbidity burdens. Future research should include larger, multicenter cohorts with protocolized imaging, blinded interpretation, longitudinal DV surveillance, adjusted analyses, and standardized neurodevelopmental assessments to validate these findings and refine thresholds for intervention and timing of delivery in pregnancies complicated by FGR.

## Conclusions

This study evaluated whether DV Doppler velocimetry predicts adverse perinatal outcomes in pregnancies complicated by FGR and whether DV findings can guide the timing of delivery. The analysis examined associations between DV Doppler status and fetal acid-base balance, Apgar scores at routine early postnatal assessments, neonatal morbidity, and neurological outcomes in early and later infancy.

A pathological DV waveform, absent or reversed A-wave, was strongly associated with fetal acidosis, low Apgar scores, neonatal morbidity, and neurological injury, whereas a normal DV waveform aligned with reassuring acid-base status and favorable clinical outcomes. These results indicate that DV Doppler adds clinically actionable information beyond arterial Doppler, offering high specificity with moderate sensitivity and enabling meaningful risk stratification in FGR. Incorporating DV evaluation into routine surveillance can prompt timely delivery when venous compromise is present and support expectant management when venous flow remains normal, helping clinicians balance the risks of prematurity against progressive hypoxic-ischemic injury and may inform perinatal care pathways.
